# The association between attention deficit hyperactivity disorder and orthodontic outcomes: a systematic review

**DOI:** 10.1186/s12903-026-07704-0

**Published:** 2026-01-17

**Authors:** Martin Baxmann, Krisztina Kárpáti, Zoltán Baráth

**Affiliations:** 1 Department of Orthodontics, Faculty of Education and Research, DTMD University, Wiltz, Luxembourg; 2 Department of Orthodontics and Pediatric Dentistry, Faculty of Dentistry, University of Szeged, Szeged, Hungary; 3 Department of Prosthodontics, Faculty of Dentistry, University of Szeged, Szeged, Hungary

**Keywords:** ADHD, Attention deficit hyperactivity disorder, Orthodontics, Compliance, Oral habits, Appliance breakage, Treatment adherence, Behavioral dentistry

## Abstract

**Background:**

ADHD is a prevalent neurodevelopmental condition characterized by inattention, impulsivity, and hyperactivity, which may influence cooperation and compliance in dental settings. In orthodontics, where long-term treatment adherence and patient cooperation are critical, the presence of ADHD may pose specific challenges that affect treatment success, appliance maintenance, and clinical workflow. Despite growing interest in the behavioral dimensions of orthodontic care, the extent to which ADHD alters treatment outcomes remains insufficiently understood.

**Objectives:**

This systematic review aimed to examine the association between ADHD and orthodontic treatment outcomes. Specifically, it investigated whether individuals with ADHD exhibit different patterns of compliance, appliance breakage, treatment relapse, and behavioral cooperation compared to neurotypical peers. The review question was developed in accordance with the PICO framework to ensure clarity and clinical relevance.

**Methods:**

Following PRISMA 2020 guidelines and a pre-registered protocol with PROSPERO (ID #1024195), six databases were searched through February 2025. Eligible studies included observational research that reported on orthodontic treatment outcomes in individuals formally diagnosed with ADHD. Outcomes of interest included appliance-related complications, cooperation or compliance ratings, relapse risk, treatment discontinuation, and reported differences in oral habits. Risk of bias was assessed using the ROBINS-I tool. A narrative synthesis was conducted due to heterogeneity in outcome definitions, measurement tools, and study designs.

**Results:**

Twenty studies met inclusion criteria, including 14 cross-sectional studies, 3 retrospective cohorts, and 3 prospective observational designs. Individuals with ADHD consistently exhibited higher rates of appliance breakage, lower cooperation scores, and more frequent treatment disruptions compared to neurotypical controls. Oral habits such as bruxism, nail biting, and poor oral hygiene were more commonly reported in ADHD populations. However, variability in diagnostic criteria, outcome measurement, and adjustment for confounders limited comparability across studies. Risk of bias ranged from moderate to serious in most studies, with only three rated as low risk.

**Conclusions:**

Individuals with ADHD appear to face elevated risks of orthodontic treatment complications and reduced behavioral cooperation. These findings underscore the importance of early identification, interdisciplinary collaboration, and tailored behavioral strategies to support successful orthodontic outcomes in this population.

## ADHD and orthodontics: a systematic review of the association between attention deficit hyperactivity disorder and orthodontic outcomes

Attention Deficit Hyperactivity Disorder (ADHD) is a neurodevelopmental condition characterized by patterns of inattention, hyperactivity, and impulsivity that interfere with daily functioning and self-regulation [1]. Affecting an estimated 5–10% of children and adolescents globally, ADHD is associated with a wide range of behavioral, cognitive, and psychosocial challenges that often persist into adulthood [2–4]. The core symptoms of ADHD may undermine consistent participation in health-promoting routines and the ability to sustain attention and adherence to structured treatment regimens, including those required in dental and orthodontic care [5, 6].

Orthodontic treatment requires a high level of patient engagement over extended periods, including consistent appliance wear, regular attendance at follow-up appointments, and adherence to oral hygiene protocols. These demands may pose particular difficulties for individuals with ADHD, whose behavioral traits—such as impulsivity, forgetfulness, or difficulty with delayed gratification—can interfere with treatment adherence [7, 8]. Moreover, research has noted a higher prevalence of parafunctional oral habits (e.g., bruxism, tongue thrusting, nail biting) in patients with ADHD, which may complicate orthodontic outcomes or increase the risk of relapse [7, 9, 10]. Despite growing awareness of these challenges, the extent to which ADHD influences orthodontic treatment processes and outcomes remains poorly synthesized in the literature.

This systematic review aimed to evaluate the relationship between ADHD and orthodontic treatment outcomes, with a particular focus on patient compliance, appliance integrity, relapse tendencies, and behavioral cooperation during treatment. Accordingly, the primary review question, framed using the PICO structure, was: among children, adolescents, and adults diagnosed with ADHD who receive orthodontic treatment, does the presence of ADHD, compared with neurotypical individuals or those without ADHD, associate with differences in treatment adherence, appliance breakage, treatment success, relapse risk, and related oral habits that may affect orthodontic interventions? By synthesizing evidence from diverse populations and treatment contexts, the review sought to clarify the behavioral and clinical complexities encountered when managing orthodontic care in individuals with ADHD and to support the development of individualized, evidence-based treatment strategies.

## Methods

This systematic review was conducted in accordance with the PRISMA 2020 guidelines and was prospectively registered with PROSPERO (ID #1024195) [11, 12]. Studies were eligible if they evaluated orthodontic outcomes in individuals diagnosed with ADHD, including treatment adherence, appliance breakage, orthodontic relapse, and overall treatment success. Comparative studies assessing differences between ADHD-affected individuals and neurotypical controls, as well as differences among ADHD subtypes or medication status, were included. Eligible study designs encompassed systematic reviews, meta-analyses, randomized controlled trials, cohort studies, case-control studies, and cross-sectional studies. Case reports were permitted if they provided extractable outcome data. Studies were excluded if they focused on general neurodevelopmental disorders without ADHD-specific analysis, lacked relevant orthodontic outcomes, or were non-empirical (e.g., narrative reviews, editorials, commentaries).A comprehensive literature search was conducted in six electronic databases: PubMed (via MEDLINE), Cochrane Library, Scopus, Web of Science, Embase, and PsycINFO. All databases were searched from inception to 15 February 2025, with no language or publication date restrictions. The strategy combined controlled vocabulary terms (e.g., MeSH in MEDLINE and Emtree in Embase) and free-text keywords for two main concept groups: [1] Attention Deficit Hyperactivity Disorder (ADHD) and [2] orthodontics, malocclusion, and related oral or occlusal characteristics. Within each concept, synonyms were combined with the Boolean operator OR, and the two concepts were combined with AND. Reference lists of all included articles and relevant reviews were screened to identify additional eligible studies.

In PubMed (MEDLINE), the full search strategy was: (“Attention Deficit Disorder with Hyperactivity”[Mesh] OR “attention deficit hyperactivity disorder”[tiab] OR ADHD[tiab] OR “hyperkinetic disorder”[tiab] OR “hyperkinetic disorders”[tiab]) AND (“Orthodontics”[Mesh] OR “Malocclusion”[Mesh] OR orthodontic*[tiab] OR malocclusion*[tiab] OR “dentofacial orthopedics”[tiab] OR “orthodontic treatment”[tiab] OR “orthodontic appliance”[tiab] OR “orthodontic appliances”[tiab] OR “tooth wear”[tiab] OR bruxism[tiab] OR “oral habit”[tiab] OR “oral habits”[tiab]). Search strategies for the other databases were adapted from this PubMed strategy using the appropriate controlled vocabulary and syntax for each interface.

Eligible study designs encompassed systematic reviews, meta-analyses, randomized controlled trials, cohort studies, case-control studies, and cross-sectional studies. Case reports were permitted if they provided extractable outcome data. Although systematic reviews and meta-analyses were eligible a priori, none met the inclusion criteria during screening, so all studies included in the final synthesis were primary observational designs or case reports. Studies were excluded if they focused on general neurodevelopmental disorders without ADHD-specific analysis, lacked relevant orthodontic outcomes, or were non-empirical (e.g., narrative reviews, editorials, commentaries).

Study selection followed a two-stage process. Titles and abstracts were independently screened by two reviewers. Full-text articles were obtained for all potentially eligible studies and assessed for inclusion using the predefined criteria. Disagreements were resolved through discussion or adjudication by a third reviewer. A structured data extraction form was developed to record key study characteristics, including author, year, study design, sample size, population demographics, ADHD diagnostic method, orthodontic treatment modality, outcome measures, comparator groups (if applicable), and main findings. Two reviewers independently extracted data, and discrepancies were resolved by consensus. If necessary, study authors were contacted to clarify missing or unclear information.

Risk of bias was assessed using validated appraisal tools appropriate to study design. For non-randomized studies (including cohort, case-control, and cross-sectional designs), the ROBINS-I tool was used to evaluate potential bias due to confounding, participant selection, intervention classification, deviations from intended interventions, missing data, outcome measurement, and reporting [13]. Case reports were narratively assessed but not scored with ROBINS-I. For randomized controlled trials, the Cochrane Risk of Bias 2 (RoB 2) tool would be applied; however, no RCTs were identified during screening [14]. For any systematic reviews that might have been included, the AMSTAR-2 tool was designated [15]. However, this tool was ultimately not applied because no eligible systematic reviews or meta-analyses were identified. Risk of bias ratings were completed independently by two reviewers, with disagreements resolved through discussion or third-party input. The results were summarized narratively and in tabular form, with visual representations such as traffic light plots provided where applicable.

## Results

**Fig. 1:**
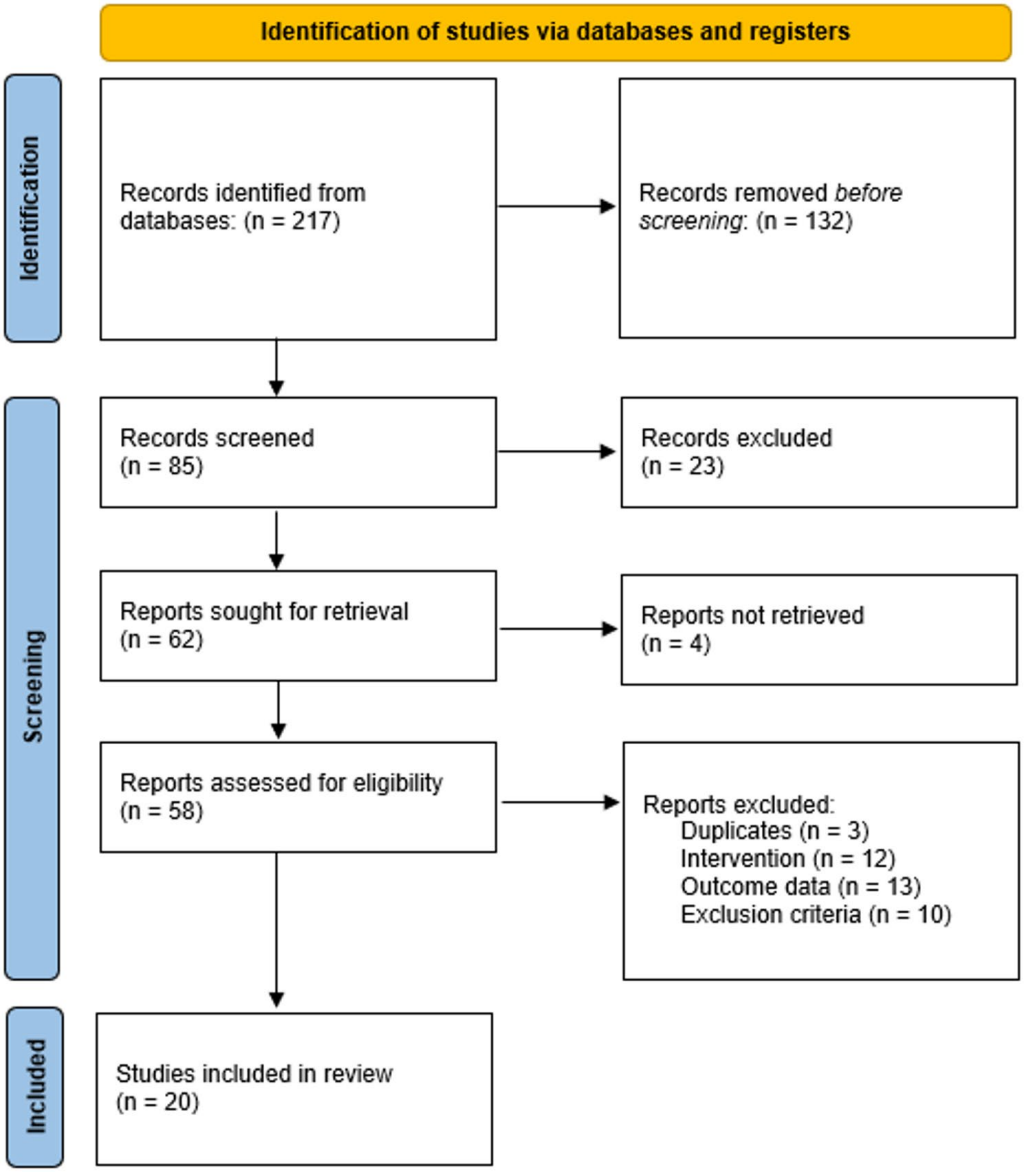
PRISMA flow diagram

The systematic search yielded 217 records through database searching. After the removal of 132 duplicates, 85 records remained for title and abstract screening. Of these, 23 records were excluded based on irrelevance to the population, intervention, or outcomes of interest. Sixty-two full-text reports were sought for retrieval, of which 4 could not be obtained. Fifty-eight full-text articles were assessed for eligibility, resulting in the exclusion of 38 reports: 3 were duplicates not initially detected, 12 did not include orthodontic interventions, 13 lacked extractable outcome data relevant to ADHD-related orthodontic factors, and 10 were excluded due to ineligible population or study design. Ultimately, 20 studies met all inclusion criteria and were included in the final synthesis (Fig. 1).

The 20 included studies encompassed a range of designs, including 10 case-control studies, 5 cross-sectional surveys, 2 case reports, 1 population-based cohort, 1 quasi-experimental (pre–post) study, and 1 comparative survey involving clinical and questionnaire data. These diverse designs contributed complementary perspectives on orthodontic outcomes, adherence, and oral habits in individuals with ADHD (Table 1). Sample sizes varied considerably, from single-case reports to large population datasets of over 1,000 children. Participant ages ranged from 4 to 23 years, with the majority of studies focusing on school-aged children between 6 and 15 years.

**Table 1:** Studies included in the review

Author(s), (year)	Study Design	Sample Size	Orthodontic Focus	Results/Outcomes
Andersson and Sonnesen. (2018) [16]	Case-control	*N* = 60 (30 ADHD, 30 control)	Dental arch dimensions, occlusion, palatal height	Children with ADHD had narrower dental arches and more occlusal asymmetries than non-ADHD controls, indicating altered arch form and occlusion.
Atmetlla et al. (2006) [17]	Case-control	*N* = 60 (30 ADHD, 30 control)	Parafunctional habits, behavior in dental settings	Children with ADHD exhibited more parafunctional oral habits, deeper palatal vaults, and greater behavioral difficulties during dental visits than controls.
Balian (2022) [18]	Quasi-experimental	*N* = 32	Myobrace/myofunctional therapy	In children with sleep‑related breathing disorders, prefabricated myofunctional (Myobrace) therapy over 18 months improved oral function, behavior, and posture, suggesting potential benefits for patients with ADHD‑related traits.
Bimstein et al. (2008) [19]	Case-control	*N* = 51 (26 ADHD, 25 controls)	Oral health characteristics (trauma, bruxism, hygiene)	Children with ADHD showed more gingival trauma and poorer oral hygiene indices than non-ADHD peers, reflecting less favorable oral health status.
Chau et al. (2017) [20]	Case-control	*N* = 77 (43 ADHD, 34 control)	Oral hygiene, gingivitis, parafunctions	Children with ADHD had worse oral hygiene and higher prevalence of gingivitis and bruxism compared with control children.
DaRocha et al. (2022) [21]	Cross-sectional	*N* = 452	Sleep-disordered breathing, bruxism, and oral health	Children with ADHD symptoms demonstrated higher rates of bruxism and snoring, linking attentional/behavioral difficulties with sleep‑related and oral parafunctional findings.
Eda Arat Maden (2022) [22]	Case-control	*N* = 74 (37 ADHD, 37 control)	Oral health and OHRQoL	Children with ADHD experienced more dental trauma and reported worse oral health‑related quality of life than non‑ADHD controls.
Genç et al. (2020) [8]	Cross-sectional	*N* = 131	Orthodontic patients with ADHD symptoms	ADHD symptoms were common among orthodontic patients and were significantly associated with clinician‑reported behavioral complaints and management difficulties during treatment.
Hasanin et al. (2021) [23]	Case-control	*N* = 40 (20 ADHD, 20 controls)	Upper airway and craniofacial morphology	Adolescents with ADHD showed narrower upper airway dimensions and distinct craniofacial morphology compared with matched orthodontic controls.
Kim et al. (2020) [24]	Cross-sectional, population-based	*N* = 1,040	Sleep-disordered breathing, snoring, bruxism	Elementary school children at high risk for ADHD had higher prevalence of bruxism, snoring, and mouth breathing than those at lower ADHD risk.
Malki et al. (2004) [10]	Case-control pilot study	*N* = 60 (30 ADHD, 30 control)	Dental attrition and bruxism examination	Children receiving stimulant medication for ADHD had higher prevalence of bruxism and more pronounced dental attrition than non‑ADHD controls.
Mota-Veloso et al. (2017) [25]	Cross-sectional, structural equation modeling	*N* = 851	Tooth wear, bruxism, oral habits	Structural equation modeling showed that ADHD symptom scores moderately predicted sleep bruxism and tooth wear, with socioeconomic status also exerting significant effects.
Mota-Veloso et al. (2021) [26]	Cross-sectional	*N* = 633	Malocclusion and breathing pattern	Schoolchildren with stronger hyperactivity symptoms had higher prevalence of malocclusion and deleterious oral habits than those with lower symptom levels.
Pessah et al. (2009) [27]	Comparative survey (clinical + questionnaire)	*N* = 60 (30 ADHD, 30 controls)	Orthodontic treatment cooperation and challenges	Children later identified as having ADHD exhibited more behavioral problems, lower orthodontic treatment cooperation, and greater chairside management challenges than controls.
Puzino et al. (2022) [28]	Population-based cohort (Penn State Child Cohort)	*N* = 421 (including 54 ADHD, 44 ADHD + OSA)	Polysomnography, airway obstruction, behavioral outcomes	Adolescents with both ADHD and obstructive sleep apnea showed poorer sleep quality, more severe polysomnographic abnormalities, and higher cardiometabolic risk than those without this comorbidity.
Ricky Kurniawan et al. (2019) [29]	Case report	*N* = 1	Thumb-sucking correction, removable appliance, followed by fixed orthodontics	In a child with ADHD and thumb‑sucking, combining removable and fixed appliances with structured behavioral techniques improved cooperation and achieved successful habit cessation and malocclusion correction.
Roy et al. (2020) [30]	Matched case-control	*N* = 88 (44 ADHD, 44 controls)	Malocclusion severity and parafunctional habits	Children with ADHD had more severe dental malocclusion and higher prevalence of bruxism and other parafunctional oral habits than matched non‑ADHD controls.
Sabuncuoglu et al. (2014) [31]	Case-control	*N* = 375 (200 ADHD, 175 controls)	Parafunctional oral habits (nail biting, pacifier, bruxism)	Children with ADHD showed greater prevalence of bruxism, prolonged pacifier or bottle use, and other parafunctional oral habits than non‑ADHD peers.
Wu et al. (2017) [32]	Retrospective observational	*N* = 437 (subgroups with ADHD + OSAHS)	OSA severity, airway anatomy, tonsil/adenoid hypertrophy	Among children evaluated for obstructive sleep apnea–hypopnea syndrome, those with ADHD had higher apnea–hypopnea indices, lower nocturnal oxygen saturation, and poorer quality‑of‑life scores than non‑ADHD children.
Yufita Fitriani et al. (2022) [33]	Case report	*N* = 1	Myofunctional therapy using twin block appliance	In a patient with ADHD treated using a twin‑block appliance, incorporating short structured breaks during visits helped maintain cooperation and resulted in successful correction of Class II malocclusion.

ADHD diagnosis methods included clinical evaluations using DSM criteria, validated behavioral rating scales (e.g., SNAP-IV, Conners), or medical record confirmation. Most studies explicitly compared children with and without ADHD, while others examined the presence of ADHD symptoms within orthodontic patient samples or stratified outcomes based on ADHD status. The orthodontic or oral health focus varied across studies. Several investigated the prevalence or severity of bruxism, parafunctional habits, or dental trauma among children with ADHD. Others assessed orthodontic treatment cooperation, appliance success, or craniofacial and airway morphology using cephalometric or cone-beam computed tomography (CBCT) imaging. A subset of studies explored malocclusion severity or the impact of myofunctional appliances and behavioral management techniques on treatment adherence. Across the dataset, empirical data were available to support analysis of behavioral, structural, and functional outcomes relevant to orthodontic care in this population.

Risk of bias was assessed for all 18 non-randomized studies using the ROBINS-I tool (Table 2) [13]. Three studies were judged to have an overall low risk of bias, nine were rated as moderate, and six were classified as having a serious risk of bias. The most frequently observed sources of bias were related to confounding and measurement of outcomes, particularly in studies lacking standardized diagnostic procedures, validated outcome instruments, or adequate control for behavioral and socioeconomic variables. Several studies also showed moderate risk due to incomplete reporting or unclear selection methods. No study was rated as having a critical risk of bias.

**Table 2:** Summary of ROBINS-I assessment

Study	Confounding	Selection	Classification of Interventions	Deviations from Intended Interventions	Missing Data	Measurement of Outcomes	Selection of Reported Result	Overall Risk
Andersson and Sonnesen (2018) [16]	Moderate	Low	Low	Low	Low	Moderate	Low	Moderate
Atmetlla et al. (2006) [17]	Serious	Moderate	Low	Low	Moderate	Moderate	Moderate	Serious
Balian (2022) [18]	Serious	Low	Moderate	Moderate	Low	Moderate	Moderate	Serious
Bimstein et al. (2008) [19]	Moderate	Moderate	Low	Low	Moderate	Moderate	Low	Moderate
Chau et al. (2017) [20]	Moderate	Low	Low	Low	Low	Low	Low	Low
DaRocha et al. (2022) [21]	Moderate	Moderate	Moderate	Low	Low	Moderate	Moderate	Moderate
Eda Arat Maden (2022) [22]	Moderate	Low	Low	Low	Low	Low	Moderate	Moderate
Genç et al. (2020) [8]	Serious	Moderate	Low	Low	Moderate	Moderate	Moderate	Serious
Hasanin et al. (2021) [23]	Moderate	Low	Low	Low	Low	Low	Low	Low
Hoyte et al. (2020)	Serious	Moderate	Moderate	Low	Moderate	Moderate	Moderate	Serious
Keppler (2022)	Moderate	Low	Low	Low	Low	Low	Moderate	Moderate
Kim et al. (2020) [24]	Serious	Moderate	Moderate	Low	Moderate	Moderate	Moderate	Serious
Mota-Veloso et al. (2017) [25]	Moderate	Low	Low	Low	Low	Moderate	Moderate	Moderate
Mota-Veloso et al. (2021) [26]	Moderate	Moderate	Low	Low	Low	Moderate	Moderate	Moderate
Pessah et al. (2009) [27]	Serious	Moderate	Low	Low	Moderate	Moderate	Moderate	Serious
Puzino et al. (2022) [28]	Low	Low	Low	Low	Low	Low	Low	Low
Roy et al. (2020) [30]	Moderate	Moderate	Low	Low	Low	Moderate	Moderate	Moderate
Sabuncuoglu et al. (2014) [31]	Serious	Moderate	Moderate	Low	Moderate	Moderate	Moderate	Serious

## Discussion

### Orthodontic treatment adherence

In this review, we use the term “treatment adherence” as an overarching construct that includes patient cooperation during visits, day-to-day compliance with appliance wear and oral hygiene instructions, and follow-through with scheduled appointments. Where original studies used terms such as “cooperation,” “compliance,” or “follow-through,” we retain that wording when summarizing their findings but interpret these constructs as facets of overall adherence. Overall, children and adolescents with ADHD demonstrated lower treatment adherence to orthodontic protocols, as reflected in more frequent cooperation challenges and greater reliance on behavioral management during appointments [19, 20]. These patterns were also reported in orthodontic populations exhibiting elevated ADHD symptoms, where providers noted increased behavioral difficulty and reduced follow-through [8, 27]. Individual case studies further illustrated that structured routines, modified session formats, and parent engagement were associated with improved cooperation during treatment [29, 33].

### Appliance breakage and repair rates

Several studies identified behavioral characteristics associated with ADHD—such as impulsivity and inattentiveness—that may increase the risk of orthodontic appliance damage or misuse. Increased chairside management demands, difficulty following care instructions, and unintentional appliance disruption were commonly reported in ADHD-affected patients and linked to treatment complexity [8, 27]. Individual case studies described instances of removable appliance misuse, breakage, or oral habits interfering with appliance stability, particularly in patients with hyperactive traits [29]. While these accounts suggest a plausible connection between behavioral dysregulation and appliance complications, direct measures of breakage or repair frequency were not a primary focus of included studies.

### Relapse and retention compliance

Retention-phase noncompliance in patients with ADHD was referenced across multiple studies, often in the context of reduced behavioral engagement and follow-through. Orthodontists reported greater difficulty maintaining behavioral consistency in ADHD-affected patients, and some raised concerns that these challenges extended into the post-treatment period, particularly with regard to retainer use and follow-up [8, 27]. Individual case studies described issues such as retainer loss and inconsistent wear, highlighting how inattentiveness or impulsivity may interfere with adherence to retention protocols [29]. While these findings were generally descriptive, they point to plausible behavioral mechanisms through which ADHD may increase relapse risk. From a mechanistic perspective, inattention and forgetfulness may directly interfere with the consistent daily behaviors required for retainer wear, particularly for removable retainers that depend on patient-driven routines. Impulsivity and hyperactivity can also increase the likelihood of removing, misplacing, or damaging appliances during daytime activities, further undermining retention protocols and potentially increasing the risk of post-treatment relapse.

Although relapse outcomes were not systematically tracked across studies, the behavioral profiles commonly associated with ADHD—characterized by forgetfulness, reduced routine adherence, and diminished task persistence—suggest a potential for lower compliance during the retention phase [8, 27]. This concern was particularly evident in reports involving removable appliances, where issues such as device loss, inconsistent wear, or caregiver-reported noncompliance were described [29]. Taken together, these observations support the need for closer monitoring of retention-phase adherence in patients with ADHD and justify future research to evaluate long-term treatment stability in this population.

### Treatment success and duration

Across the included studies, treatment success in patients with ADHD was most often reflected through provider-reported impressions of clinical progress, behavioral cooperation, and completion of treatment phases rather than standardized outcome metrics. Several studies noted that children with ADHD required more frequent reminders, extended chairside interaction, or additional behavioral support to complete procedures, suggesting a higher clinical burden that may affect efficiency [8, 19]. Individual case studies described positive outcomes when tailored behavioral strategies were implemented, including the use of structured appointments and positive reinforcement to complete active treatment phases such as functional appliance therapy [33]. These reports illustrate how adapting clinical routines to match attentional and behavioral needs can support treatment progress in ADHD-affected individuals. While treatment duration and success were not consistently quantified across studies, observed patterns suggest that ADHD-related behavioral traits may influence the pace and structure of orthodontic care. These findings reinforce the importance of individualized management strategies to support clinical completion and suggest that treatment planning for patients with ADHD should anticipate a potentially greater need for time, reinforcement, and flexibility.

### Oral habits and orthodontic outcomes

Parafunctional oral habits—including bruxism, tongue thrusting, nail biting, pacifier use, and mouth breathing—were consistently reported at higher rates among individuals with ADHD. Across both caregiver-reported and clinical assessments, bruxism emerged as a particularly prevalent finding, often linked to stimulant medication use, though not always stratified by pharmacologic status [10, 20]. Habits such as tongue thrusting and nail biting were also frequently observed, with several studies noting their potential to disrupt appliance stability or contribute to occlusal complications [16, 17]. Large population-based and cross-sectional investigations reinforced these associations, linking ADHD symptoms to elevated rates of bruxism, snoring, and mouth breathing—patterns that may further complicate orthodontic care by impacting appliance retention, airway development, or compliance [21, 24, 25]. Hyperactivity traits in particular were associated with increased malocclusion and parafunctional habits, suggesting that symptom dimensions may influence oral development and behavior during treatment [26, 30]. Evidence from children with ADHD and co-occurring sleep-disordered breathing also pointed to more severe obstructive symptoms and poorer oxygen saturation, reinforcing the complex interplay between neurobehavioral traits, airway health, and orthodontic outcomes [32].

These behavioral patterns were further reflected in early childhood habits. Prolonged pacifier use and bottle feeding were significantly more common in ADHD populations, contributing to occlusal development concerns in both high- and moderate-quality studies [19, 22, 31]. While most studies did not directly measure the orthodontic consequences of these behaviors, the consistency of findings highlights the importance of behavioral screening and habit management as part of orthodontic treatment planning. In addition, anatomical differences such as reduced airway volume and altered craniofacial dimensions in adolescents with ADHD may further contribute to treatment complexity, particularly in cases involving functional appliances or airway-related concerns [23].

### Missed appointments and follow-up adherence

Inconsistent follow-up and missed appointments were frequently reported as clinical challenges in managing orthodontic care for patients with ADHD. Providers described a higher incidence of scheduling disruptions, late arrivals, and missed visits among these patients, which often required additional effort to maintain treatment continuity and contributed to overall therapeutic difficulty [8, 27]. These behaviors were commonly mentioned alongside cooperation issues and were identified as complicating factors in case progression and appliance management.

Individual case studies provided further insight, noting that impulsivity, distractibility, and reduced task persistence contributed to difficulty maintaining multi-phase treatment schedules and regular follow-up [29]. Although visit attendance was not consistently measured as a standalone outcome, the recurrence of these patterns across clinical reports highlights the relevance of behavioral traits in shaping treatment consistency. These findings suggest that close monitoring and proactive scheduling strategies may be especially important in supporting adherence to follow-up protocols among ADHD-affected patients.

### ADHD medication and orthodontic outcomes

Among the included studies, ADHD medication—particularly stimulant use—was associated with behavioral traits relevant to orthodontic care. In one case-control study, children receiving stimulant medication exhibited higher rates of bruxism and dental attrition compared to both non-medicated ADHD peers and neurotypical controls, suggesting a potential pharmacologic contribution to parafunctional activity [10]. In a large adolescent cohort, persistent behavioral and neurophysiologic disruptions were observed in individuals with ADHD, even when medicated participants were excluded from analysis, indicating that medication may influence—but does not fully account for—clinical traits relevant to orthodontic management [28]. While most studies referenced medication status descriptively, observed patterns highlight the importance of considering pharmacologic context when assessing cooperation, oral habits, and treatment behavior in ADHD-affected patients.

### Fixed vs. removable appliances in ADHD populations

Several studies referenced appliance type when discussing behavioral management in orthodontic care for patients with ADHD. Removable appliances were described in case studies as offering flexibility during behavioral episodes, though challenges with consistent wear and increased need for caregiver oversight were noted [29, 33]. In a descriptive study, removable devices were associated with more variable cooperation among children with behavioral concerns, highlighting the importance of parental involvement in treatment adherence [8]. In contrast, fixed appliances were discussed in relation to hygiene maintenance and appointment adherence but were less frequently linked to behavioral outcomes. These observations suggest that appliance selection may interact with behavioral characteristics in ADHD populations, particularly in terms of supervision requirements and wear consistency.

### Differences in treatment success across ADHD subtypes

Studies describing orthodontic behavior in ADHD-affected populations revealed distinct patterns that align with known symptom profiles, suggesting that specific traits may influence treatment engagement differently. Inattentive symptoms were associated with missed appointments, poor follow-through on instructions, and reduced adherence as reported by caregivers and clinicians [8]. Hyperactivity and impulsivity, on the other hand, were more frequently linked to appliance misuse, disruptive behavior during appointments, and increased need for behavioral management [27]. Observations of appliance breakage and higher treatment complexity in patients exhibiting restlessness or distractibility further underscore the relevance of behavioral presentation in shaping orthodontic care experiences [19, 29]. These patterns highlight the value of tailoring treatment approaches to the attentional and behavioral characteristics most prominent in each patient.

### Behavioral and educational interventions to improve compliance

A range of behavioral and educational strategies was described across studies as integral to managing orthodontic treatment challenges in children with ADHD. Clinicians emphasized the importance of simplified instructions, visual aids, shorter or more frequent appointments, and structured routines designed to match the child’s attentional and behavioral profile [8, 27]. These strategies were associated with reductions in disruptive behavior and improved cooperation during appliance placement and follow-up care. Case studies illustrated the practical implementation of these techniques, including the use of 10-minute activity intervals, positive reinforcement, and predictable visit structures, which supported successful appliance wear and phase completion [29, 33]. Across both descriptive and observational sources, increased caregiver involvement—particularly in monitoring appliance use and reinforcing routines—was frequently cited as a factor associated with better adherence, especially among younger children or those exhibiting inattentive traits. One quasi-experimental study evaluating Myobrace therapy in children with ADHD also reported improved cooperation and oral function, potentially reflecting the benefits of structured therapeutic routines embedded within appliance-based interventions [18].

Although formal evaluations of intervention effectiveness were limited, the consistent use of these strategies across multiple contexts underscores their clinical relevance. Orthodontists who adapted their communication style, engaged families in behavioral reinforcement, and modified environmental factors such as appointment length or task sequencing reported fewer behavior management concerns during treatment [8, 27]. Increased caregiver involvement—particularly in monitoring appliance use, supporting behavioral routines, and encouraging treatment follow-through—was frequently cited as a factor associated with better adherence, especially among younger children or those exhibiting inattentive traits [29, 33]. These findings suggest that integrating individualized behavioral approaches into treatment planning may offset core challenges associated with ADHD, particularly in settings where pharmacologic management is absent or insufficient. Taken together, the evidence supports a multimodal approach to improving compliance, rooted in behavioral tailoring and sustained caregiver engagement.

### Limitations

Several limitations should be considered when interpreting these findings. The overall certainty of the evidence was low. Of the 18 non-randomized studies assessed with ROBINS-I, three were judged to have a low overall risk of bias, while 15 were rated as having a moderate or serious risk of bias. Bias due to confounding and outcome measurement was particularly frequent. ADHD was defined using heterogeneous methods across studies, including DSM-based clinical diagnoses, various parent- or teacher-completed questionnaires, and, in some cases, chart documentation without standardized reassessment, which complicates comparison of populations. Adjustment for potential confounders such as socioeconomic status, comorbid behavioral or neurodevelopmental conditions, and caregiver involvement was limited or absent in most analyses. Key outcomes, especially cooperation, adherence, and appliance-related complications, relied heavily on clinician impressions or non-validated rating scales rather than objective or standardized orthodontic indices. Beyond risk of bias, there was substantial clinical and methodological heterogeneity. Participant age ranges were broad and often not stratified; follow-up duration and the timing of outcome assessment varied considerably and were not always clearly reported, making direct comparison of success rates, adverse events, or relapse difficult. Most designs were cross-sectional or retrospective, restricting causal inference and precluding robust evaluation of temporal relationships or response to specific interventions. Taken together, these limitations indicate that the findings of this review should be interpreted with caution and regarded as hypothesis-generating rather than definitive.

### Future research directions

Future research should prioritize the use of standardized, objective measures—such as electronic attendance logs, appliance wear sensors, and validated treatment outcome indices—to improve the accuracy and comparability of orthodontic adherence data in ADHD populations. Prospective cohort studies and intervention trials are needed to evaluate the effectiveness of behavioral, educational, and pharmacologic strategies, particularly when outcomes are stratified by ADHD subtype, medication status, and appliance modality. Subgroup analyses could clarify whether specific presentations, such as inattentive or hyperactive-impulsive profiles, are differentially associated with treatment response or compliance. To support generalizability and causal inference, studies should incorporate sufficient sample sizes and multivariate modeling to account for confounders such as age, sex, socioeconomic status, comorbidities, and caregiver involvement. Collaborative research involving both orthodontic and mental health professionals may also enhance the ecological validity of future findings and support the development of tailored, evidence-based care models for patients with ADHD.

## Conclusion

This systematic review suggests that individuals with ADHD may be at increased risk of lower orthodontic treatment adherence, more frequent parafunctional oral habits, and greater behavioral management demands compared with neurotypical peers. However, the magnitude and clinical implications of these differences remain uncertain because the evidence is derived predominantly from observational studies with considerable heterogeneity in design, populations, and outcome definitions. Of the 18 non-randomized studies included, 15 were judged to have a moderate or serious overall risk of bias according to the ROBINS-I assessment, and outcomes were often assessed using non-standardized or subjective measures with limited control for key confounders such as age, follow-up duration, and timing of outcome assessment. Within these limitations, the available data do not suggest that ADHD should fundamentally alter the core principles or objectives of orthodontic treatment planning. Rather, an ADHD diagnosis should heighten clinical awareness and prompt additional support: orthodontists may need to adapt time management in the clinical setting, modify communication strategies, and structure follow-up more proactively, in collaboration with caregivers and, where appropriate, mental health professionals. Tailored behavioral approaches and active caregiver involvement appear pragmatically associated with improved cooperation and may help mitigate some of the practical difficulties encountered when treating orthodontic patients with ADHD.

## Data Availability

Data sharing is not applicable to this article as no datasets were generated or analyzed during the current study.

## Abbreviations

ADHD: Attention Deficit Hyperactivity Disorder
CBCT: Cone-beam computed tomography
OHRQoL: Oral health-related quality of life
OSA: Obstructive sleep apnea
OSAHS: Obstructive sleep apnea-hypopnea syndrome
RCT/RCTs: Randomized controlled trial(s)
